# Cytomegalovirus-Specific CD8+ T-Cells With Different T-Cell Receptor Affinities Segregate T-Cell Phenotypes and Correlate With Chronic Graft-Versus-Host Disease in Patients Post-Hematopoietic Stem Cell Transplantation

**DOI:** 10.3389/fimmu.2018.00760

**Published:** 2018-04-10

**Authors:** Thomas Poiret, Rebecca Axelsson-Robertson, Mats Remberger, Xiao-Hua Luo, Martin Rao, Anurupa Nagchowdhury, Anna Von Landenberg, Ingemar Ernberg, Olle Ringden, Markus Maeurer

**Affiliations:** ^1^Department of Laboratory Medicine, Karolinska University Hospital, Stockholm, Sweden; ^2^Center for Allogeneic Stem Cell Transplantation, Karolinska University Hospital, Stockholm, Sweden; ^3^Department of Oncology-Pathology, Karolinska Institutet, Stockholm, Sweden; ^4^Department of Microbiology, Tumor and Cell Biology, Karolinska Institutet, Stockholm, Sweden

**Keywords:** cytomegalovirus, cytomegalovirus-specific CD8+ cytotoxic T-lymphocytes, T-cell receptor affinity, T-cell, hematopoietic stem cell transplantation, programmed cell death-1, tetramers

## Abstract

Virus-specific T-cell responses are crucial to control cytomegalovirus (CMV) infections/reactivation in immunocompromised individuals. Adoptive cellular therapy with CMV-specific T-cells has become a viable treatment option. High-affinity anti-viral cellular immune responses are associated with improved long-term immune protection against CMV infection. To date, the characterization of high-affinity T-cell responses against CMV has not been achieved in blood from patients after allogeneic hematopoietic stem cell transplantation (HSCT). Therefore, the purpose of this study was to describe and analyze the phenotype and clinical impact of different CMV-specific CD8+ cytotoxic T-lymphocytes (CMV-CTL) classes based on their T-cell receptor (TCR) affinity. T-cells isolated from 23 patients during the first year following HSCT were tested for the expression of memory markers, programmed cell death 1 (PD-1), as well as TCR affinity, using three different HLA-A*02:01 CMV_NLVPMVATV_-Pp65 tetramers (wild-type, a245v and q226a mutants). High-affinity CMV-CTL defined by q226a tetramer binding, exhibited a higher frequency in CD8+ T-cells in the first month post-HSCT and exhibited an effector memory phenotype associated with strong PD-1 expression as compared to the medium- and low-affinity CMV-CTLs. High-affinity CMV-CTL was found at higher proportion in patients with chronic graft-versus-host disease (*p* < 0.001). This study provides a first insight into the detailed TCR affinities of CMV-CTL. This may be useful in order to improve current immunotherapy protocols using isolation of viral-specific T-cell populations based on their TCR affinity.

## Introduction

Cytomegalovirus (CMV) is usually present in a latent state in a majority of the human population (1, 2). Active CMV disease and reactivation are common in immunocompromised individuals, e.g., in patients with human immunodeficiency virus (HIV) infection, solid organ transplant recipients, as well as in individuals who undergo hematopoietic stem cell transplantation (HSCT) depending on the CMV-serological status of the donor and recipient (2–4). CMV mortality post-HSCT has greatly decreased over time although, CMV disease still remains a severe and life-threatening complication with an incidence around 5% (5). CMV reactivation and disease not only have a direct impact on the outcome of the transplanted individuals, but is also known to cause severe indirect effects, such as increased risk of bacterial and fungal infections (6) and increased incidence of graft-versus-host disease (GVHD) (7, 8). In contrast, positive effects of CMV infection/reactivation have also been reported, due to a promotion of graft-versus-leukemia effects by early CMV antigenemia ensuing immune responses, such as the cross-reactivity of γδ T-cells (9–12). Nonetheless, this relation remains controversial, since several studies did not observe a decreased risk of relapse concomitant with CMV reactivation (13–15).

The standard treatment for CMV management post-HSCT is supported by prophylaxis, pre-emptive regimen, and therapy using acyclovir, valganciclovir, and ganciclovir (16). As an alternative to antiviral agents, adoptive T-cell therapy (ACT) is being used as a promising treatment of infections to overcome the immunodeficiency state of patients post-HSCT. Generation and selection of CMV-specific CD8+ cytotoxic T-lymphocytes (CMV-CTL) by *ex vivo* T-cells expansion, magnetic beads, HLA multimers, and IFN-γ capture have proven their efficiency by overcoming the lack of T-cell immunity and providing long-term protective immune response (17–23). Optimization of T-cell products for ACT has been made possible by the better understanding and characterization of the mechanism and biology of immune-protection and long-lasting cellular immune responses against transformed cells and pathogens, such as CMV (24, 25). The cell number, frequency of antigen-specific T-cells, antigen-specific immune functions, as well as the maturation and differentiation status of transferred T-cells, have proven to be vital for protective immune effector functions (26–28).

Despite high efficacy in diagnostic techniques, antiviral treatments and ACT, there is still room for improving the CMV management in patients post-HSCT. To date, the T-cell receptor (TCR) affinity of CMV-CTL using tetramers has not been analyzed in patients post-HSCT. In this report, we aimed at characterizing the HLA-A*02:01-restricted CMV-CTL repertoire in peripheral blood from HSCT recipients at various time points after transplantation based on immune reactivity to the immunodominant tegument protein CMV-pp65 (29) using three MHC class I-CMV_NLVPMVATV_ peptide tetramers targeting TCRs of different affinities. We further correlate CMV-CTL frequencies with clinical events, such as CMV reactivation and GVHD post-HSCT, which may be helpful in predicting ACT outcome as well as refining cell products.

## Materials and Methods

### Patient Characteristic and HSCT Regimen

Twenty-three patients were recruited for T-cell analysis after HLA-matched HSCT, the treatment was performed at CAST, Karolinska University Hospital, Sweden (Table 1). This study was part of a larger study that prospectively recruited 262 patients post-HSCT with blood samples collected before HSCT and at 1, 2, 3, 6, 12, and 24 months post-HSCT at CAST from 2007 to 2016. IRB approval (Stockholm Ethical Committee South 2010/760-31/1) was in place and consent was obtained from each patient. Adult patients for this study were selected based on HLA-A*02:01 positive, no anti-thymocyte globulin (ATG) treatment and availability of more than four out of seven samples. Quality control based on cell count and viability excluded 11 samples. The study, therefore, included 81 samples with 12–17 samples per time points. Most of the patients received peripheral blood stem cells from siblings after a reduced intensity conditioning (RIC) regimen and chemotherapy (Table 1). Neutrophil engraftment defined by an absolute count >0.5 × 10^9^/L for three consecutive days was reached at a median of 18 days (min. 13, max. 25). Grading of GVHD was evaluated using established criteria (30). Patients with GVHD received ≥1 mg/kg/day prednisone equivalents of corticosteroids during the study as recently described (31). CMV DNAemia was routinely monitored and quantified post-HSCT by real-time PCR on whole blood (32). Patients (*n* = 3) who had a viral CMV DNAemia of more than 2,000 copies/ml were treated as previously described (33) with intravenous ganciclovir (*n* = 2) or oral valganciclovir (*n* = 1) between day 111 and 124 post-HSCT.

**Table 1:** Patients characteristics and hematopoietic stem cell transplantation regimen.

Patient age median (range)	53 (22–72)
Patient gender (M/F)	7/16

Diagnosis	
SAA	1
AML/ALL	5/4
CML	2
Lymphoma	3
MDS/MPN	6
Myeloma	2

BM/PBSC	2/21
Sibling/MUD	20/3
Donor age	49 (23–71)

TNC dose (×10^8^/kg)	14.6 (2.7–31.6)
CD34 dose (×10^6^/kg)	6.5 (2.5–11.6)

Engraftment kinetic (days)	
Neutrophils (<0.5 × 10^9^/L)	25 (13–25)
Leukocytes (>0.2 × 10^9^/L)	0 (0–22)
Platelets (30 × 10^9^/L)	13 (0–43)

GVHD prophylaxis	
CsA + MTX	15
Tac + Sir	5
PT Cy	3

Conditioning	
MAC/RIC	5/18
TBI-based/chemo	8/15

D+/R+	13/23
D+/R−	3/23
D−/R+	5/23
D−/R−	2/23

aGVHD (0/I/II/III–IV)	5/10/4/4
cGVHD (none/mild/moderate/severe)	10/8/0/5
CMV reactivation/infection	11

Relapse	4

*M, male; F, female; SAA, severe aplastic anemia; AML/ALL, acute myeloid leukemia/acute lymphoblastic leukemia; CML, chronic myeloid leukemia; MDS/MPN, myelodysplastic syndromes/myeloproliferative neoplasms; BM/PNSC, bone marrow/peripheral blood stem cell; MUD, matched unrelated donor; TNC, total nucleated cell; CD34, hematopoietic progenitor cell; GVHD, graft-versus-host disease; CsA + MTX, cyclosporine A + methotrexate; Tac + Sir, tacrolimus + sirolimus; PT Cy, post-transplant cyclophosphamide; MAC/RIC, myeloablative/reduced intensity conditioning; TBI, total-body irradiation; CMV, cytomegalovirus; MM, mismatch*.

### CMV Tetramer Construction

The anti-CMV pp65 HLA-A2*01:02_NLVPMVATV_ tetramers were constructed in-house. The heavy and light chains of the HLA-A*02:01 allele were cloned into bacterial expression vectors (pET24d+ and pHN1). A245v and q226a mutations were intro-duced into the sequence of the A*02:01 heavy chain using a site-directed mutagenesis kit (Stratagene, La Jolla, CA, USA) (34, 35). The MHC molecules were produced as previously described (36, 37). Briefly, heavy and light chains were produced in *Escherichia coli* Bl21 DE3 pLys (Invitrogen, Carlsbad, CA, USA) as inclusion bodies. They were then solubilized in an 8 M urea buffer, pH 6.5. The heavy and light chains were purified, solubilized, and folded to correct trimeric structure in 100 mM Tris-400 mM arginine-5 mM EDTA buffer, pH 8.0 together with a peptide derived from the CMV-pp65 protein (NLVPMVATV) (Peptides&Elephants GmbH, Postdam, Germany). The correctly folded MHC monomers were biotinylated and affinity-purified. Unfolded proteins that do not form MHC monomers were precipitated and were filtered away or excluded *via* the affinity purification step. Monomeric MHC class I-peptide complexes were then tetramerized and fluorescently labeled with streptavidin–phycoerythrin (PE, Life technologies, Carlsbad, CA, USA), streptavidin–phycoerythrin/Cy7 (PE/Cy7, Biolegend, San Diego, CA, USA) or streptavidin–allophycocyanin (APC, Life technologies, Carlsbad, CA, USA).

### Flow Cytometric Analysis

Peripheral blood mononuclear cells (PBMCs) were isolated over Ficoll-Hypaque gradient (GE Healthcare, Uppsala, Sweden) and frozen at −190°C in fetal bovine serum (FBS, Life technologies, Carlsbad, CA, USA) and 10% DMSO (38). PBMCs were thawed in RPMI supplemented with 10% FBS (Life Technologies, Carlsbad, CA, USA) and washed twice in PBS-0.1% FBS. One million cells were first incubated for 30 min in dark and at 20°C with a LIVE/DEAD fixable aqua dead cell stain marker (Invitrogen, Carlsbad, CA, USA) according to the manufacturer’s instructions. After a single wash with PBS, cells were incubated for 30 min at 37°C with the three different MHC HLA-A2–NLVPMVATV (CMV-pp65) class I tetramers as wild-type (wt) CMV tetramer PE/Cy7, a245v mutant tetramer APC, and q226a mutant tetramer PE. After 30 min cells were washed in PBS-0.1% FBS and then incubated at 4°C for 15 min with the following surface marker antibodies: anti-CD3 brilliant violet 570 (clone UCHT1), anti-CD4 PE/Cy5 (clone RPA-T4), anti-CD8 APC Alexa Fluor 700 (clone SK1), anti-CCR7 brilliant violet (clone G043H7), anti-CD45RA PerCP/Cy5.5 (clone HI100), anti-PD-1 APC/Cy7 (clone EH12-2H7), and anti-IL21R PE-CF594 (clone 17A12). After washing with PBS-0.1% FBS, the cells were acquired on a FACS Aria flow cytometer (BD Biosciences, Stockholm, Sweden) and analyzed using FlowJo software (Treestar Inc., Ashland, OR, USA). Due to the HSCT procedure, the T-cell number was in some instances very low; the tetramer responses and subpopu-lations were, therefore, reported only if we could detect more than 30 absolute events. High-affinity CTLs were defined as tetramer q226a-positive events. Medium-affinity CTLs were defined as tetramer a245v-positive, q226a-negative events. Low-affinity CTLs were defined as tetramer wt-positive a245v- and q226a-negative events. The gating strategy used to define tetramer reactive T-cells is shown in Figures S1A,B in Supplementary Material.

### Validation of the CMV q226 Mutant Tetramer

High-affinity HLA-A2*01:02_NLVPMVATV_ sorted CMV-CTL from an HLA-A*02:01 positive healthy individual were seeded in duplicates in 96-well plates at a density of 100,000 cells/well in RPMI medium. Prior to the addition of CMV pp65 (NLVPMVATV) peptide, cells were pre-treated 30 min at 4°C with addition of human CD8 blocking antibody clone DK25 (0.5 µg/ml, EMD Millipore, Billerica, MA, USA) known for its potent anti-CD8 blocking ability as described in Ref. (39, 40). Total fraction of CD8+ T-cells was used for comparison; isotype IgGκ antibody was used as control. After overnight incubation at 37°C, supernatants were harvested and IFN-γ production was measured by ELISA (Mabtech, Stockholm, Sweden) according to the manufacturer’s instructions.

### Data Handling and Statistical Analysis

To analyze the impact of the different CMV-CTL classes based on TCR affinity (low-, medium-, and high-affinity), frequencies were normalized as a proportion of the total number of CMV-CTL. Briefly, the proportion of CMV-CTL classes was calculated as the following: for each individual sample the addition of low-, medium-, and high-affinity CMV-CTL represented the total number of CMV-CTL (100%). The proportion of low-, medium,- and high-affinity CMV-CTL from that total number of CMV-CTL was reported for each individual sample. Data were analyzed using GraphPad Prism 6 software (La Jolla, CA, USA). Differences were analyzed using Wilcoxon matched-pairs signed rank test or Friedman test to detect differences between two matched timepoints or repeated matched time points after HSCT and Mann–Whitney *U*-test to detect differences between two groups of unpaired samples at single time points. Two-way ANOVA was used to analyze differences between two groups over time. If significant, the Tukey’s test for multiple comparisons was used to detect differences at specific time points. The significance threshold was set at 0.05.

## Results

### Cohort Characterization and T-Cell Phenotype Post-HSCT

We monitored the immune reconstitution profile of 23 HLA-A*02:01 positive patients over a period of 12 months post-HSCT. Blood samples were obtained at defined intervals after transplantation. The CMV-serological status of the donors (D) and recipients (R) was determined before HSCT. In this cohort, 56.5% of the patients (13/23) belonged to the D+R+ group, 21.7% (5/23) belonged to the D−R+ group, 13.1% (3/23) belonged to the D+R− group, and 8.7% (2/23) were characterized as D−R−. 11/23 patients, exclusively R+, had CMV reactivation post-HSCT (Table 1). The median time to onset of acute GVHD (aGVHD: 8/23, grade II–IV) and chronic GVHD (cGVHD: 13/23) was 30 (range 17–107) and 145 (range 87–965) days, respectively. 6 out of 23 patients died after HSCT (4 of them during the study time): three due to disease relapse and the other three patients due to HSCT-related complications. Two out of six patients experienced aGVHD, while one patient was diagnosed with a secondary malignancy. One patient received a boost (donor lymphocyte infusion of 10 × 10^6^ CD34+ cells/kg at day 106 post-HSCT) (see Table S1 in Supplementary Material for more information). Finally, the chimerism analysis of the CD3+ cells showed a median range of donor chimerism above 99.9% at the two last time points post-HSCT (Figure S1C in Supplementary Material).

**Figure 1:**
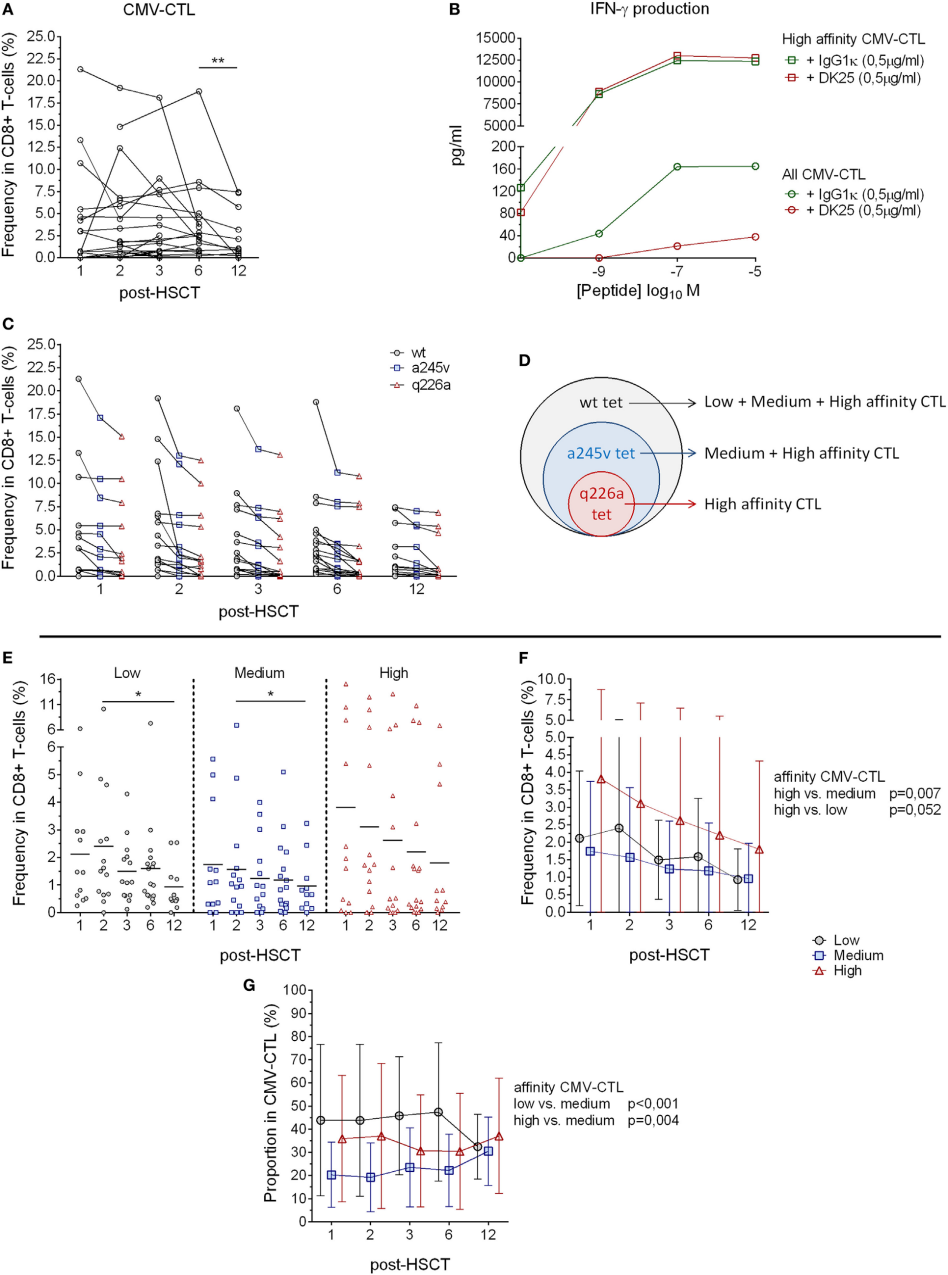
HLA-A2 cytomegalovirus (CMV) tetramer reactive CD8+ T-cells and CMV-specific T-cells post-hematopoietic stem cell transplantation (HSCT). **(A)** Analysis of the CMV-specific CD8+ cytotoxic T-lymphocytes (CMV-CTL) in 23 patients followed individually over time post-HSCT. **(B)** IFN-γ production evaluated after overnight incubation of CMV-CTL isolated by q226a mutant tetramer (square) and total fraction of CMV-CTL (circle) with different concentration of CMV peptide and CD8 blocking antibody (in red, DK25) or isotype control (green). **(C)** HLA-A2 CMV tetramer reactive CD8+ T-cells frequency post-HSCT. Dark circles represent wild-type tetramer reactive T-cells, blue squares represent a245v mutant tetramer reactive T-cells and red triangles represent q226a mutant tetramer reactive T-cells. **(D)** HLA-A2 CMV tetramer reactive CD8+ T-cells allow the analysis of the different CMV-specific CD8+ T-cells depending on the affinity of the T-cell receptor (TCR). **(E)** Frequency of CMV-CTL with different TCR affinity in peripheral blood of patient’s post-HSCT. Mean values are represented. **(F)** Analyses of the difference between the CMV-CTL subpopulations frequency in CD8+ T-cells. Mean and SD are represented. **(G)** Proportion analysis of the different subpopulations (low, medium, high-affinity) in the total of CMV-CTL. Mean and SD are represented. Black circles: low-affinity CMV-CTL, blue squares: medium-affinity CMV-CTL, red triangles: high-affinity CMV-CTL. Differences were analyzed using Wilcoxon matched-pairs signed rank test or Friedman test to detect differences between two groups of paired samples **(A,E)** and two-way ANOVA was used to detect differences between two groups over time **(F,G)**. *p < 0.05, **p < 0.01, ***p < 0.001.

The gating strategy used to characterize the CMV-CTL populations in this study is shown in Figure 1A. While we did not observe significant differences in the CD8+ T-cell population reconstitution over the five time points post-HSCT using the Friedman test, a mean increase from 32.5 to 49.6% of CD8+ T-cells and a significant increase between months 1 and 3 as well as 6 (*p* = 0.006 and *p* = 0.042, Figure S1D in Supplementary Material) was observed. CD8+ T-cell immunophenotyping based on CD45RA and CCR7 expression revealed a predominant effector memory (TEM: CD45RA− CCR7−) phenotype with a stable frequency of approximately 50% (Figure S1E in Supplementary Material). The terminally differentiated effector memory (TEMRA, Figure S1F in Supplementary Material) CD8+ T-cell population showed a dynamic change of over time post-HSCT. This subset presented an increase in frequency from 31.6 to 42.2% over time, with a statistically significant difference between months 2 and 6 post-HSCT (*p* < 0.001). Over the course of the follow-up, the low proportion of central memory (TCM: CD45RA− CCR7+) CD8+ T cells decreased from month 1 to month 12 post-HSCT (median from 2.77 to 1.31%, *p* = 0.039). We analyzed the expression and the mean fluorescence intensity (MFI) of PD-1 in CD8+ T-cell populations. Mean frequency of PD-1 expression among CD8+ T-cells ranged from 11.4 to 17.2% and the MFI for PD-1 did not differ significantly over time post-HSCT (Figures S1G,H in Supplementary Material).

To summarize, in the HLA-A*02:01+ patient cohort CD8+ T-cells exhibited predominantly a TEM phenotype, and an increased TEMRA proportion among CD8+ T cells over 12 months following HSCT.

### CMV Tetramer-Reactive CD8+ T-Cells Versus CMV-Specific CD8+ T-Cells Post-HSCT

CMV-specific CD8+ cytotoxic T-lymphocytes reconstitution after HSCT was investigated in 23 patients at 1, 2, 3, 6, and 12 months post-HSCT by tetramer analysis. A total of 13 blood samples were found negative for CMV-CTL (Figure 1A), while some patients showed a frequency up to 21.3% CMV-CTL among total CD8+ T-cells over time. We observed a decreasing CMV-CTL mean frequency from 3.9 to 2.5% between months 6 and 12 post-HSCT (*p* = 0.006), yet no significant difference was seen over time post-HSCT using the Friedman test.

To further investigate the CMV-CTL reconstitution, three different HLA-A2 tetramers presenting the same CMV_NLVPMVATV_ epitope (pp65 protein) were constructed to gauge MHC class I/CMV-specific T-cell responses (34, 35). The tetramers were (i) the wild-type tetramer; (ii) the mutant a245v tetramer, which due to the amino acid change reduces the MHC class I-CD8 co-receptor interaction (41); and (iii) the mutant q226a tetramer, which totally abrogates the interaction between MHC class I and the CD8 co-receptor (Figures S1A,B in Supplementary Material) (42–44). The q226a mutant tetramer has not been reported previously; we, therefore, validated its ability to function independently of CD8 co-receptor binding. The anti-human CD8 blocking antibody (clone DK25) is known to target CD8α and blocks the activation of CD8+ T-cells at low-affinity TCR/MHC-I interaction (39). Q226a tetramer-reactive T-cells were isolated, incubated with an anti-CD8 blocking antibody, and stimulated with CMV_NLVPMVATV_ peptide in dose-dependent manner. We did observe any inhibition of immune function detected by IFN-γ production. In contrast, total fraction of CM-CTL presented an inhibited production of IFN-γ when exposed to CD8 blocking antibody (Figure 1B). Thus, we concluded that the mutant q226a tetramer exclusively binds to high-affinity T-cells which do not require CD8 co-receptor interaction and stimulation to be activated. Wt, a245v, and q226a tetramer-reactive CD8+ T cells were present over time post-HSCT at similar proportions (Figure 1C). The wt tetramer binds to all the CMV-CTL population regardless of their TCR affinity, while the mutant a245v tetramer binds to CMV-CTL with medium and high-affinity TCR, and the mutant q226a tetramer exclusively binds to high-affinity TCR CMV-CTL (Figures 1C,D).

Cytomegalovirus tetramer-reactive CD8+ T cells do not inform on CMV-CTL affinity (Figures S1A,B in Supplementary Material). We, therefore, determined the “medium” affinity CMV-CTL as a245v positive and q226a negative, and the “low” affinity CMV-CTL as wt-positive, a245v-, and q226a-negative. The proportion of the CMV-CTL based on the TCR affinity was different from patients to patients and over time post-HSCT as some samples. In some patients, CMV-CTL with specific TCR affinity were more frequent, while other patients showed more equal proportion between the CM-CTL classes (as illustrated in Figure S2 in Supplementary Material). We observed significant differences in the frequency of low- and medium-affinity CMV-CTL between months 2 and 12 post-HSCT (*p* = 0.031 and 0.023, respectively, Figure 1E). High-affinity CMV-CTL was found to be at significantly higher frequency compared to the medium-affinity CMV-CTL (*p* = 0.007). Overall, the frequency of high- and low-affinity CMV-CTL did not differ significantly, although a trend was observed, where high-affinity CMV-CTL had a higher frequency than low-affinity CMV-CTL among CD8+ T cells (*p* = 0.052) (Figure 1F).

To further investigate the CMV-CTL classes based on the TCR affinity, we reported the proportion of the respective affinity categories (low/medium/high) of the total CMV-CTL population. While high-affinity CMV-CTL were present at higher frequencies over time post-HSCT, the medium-affinity CMV-CTL were found to be at a significant lower proportion compared to the low- and high-affinity CMV-CTL populations (*p* < 0.001 and 0.004). No differences in the proportions of high- and low-affinity CMV-CTL were observed (Figure 1G).

To summarize, mutant q226a tetramer enables the exclusive detection of CD8+ T cells with a high-affinity TCR without the requirement of the CD8− coreceptor interaction. CMV-CTL with low- and high-affinity TCRs can be found at different frequencies and proportion in patients at various time points post-HSCT.

### Effector Phenotype of the High-Affinity CD8+ CMV-Specific T-Cells

**Figure 2:**
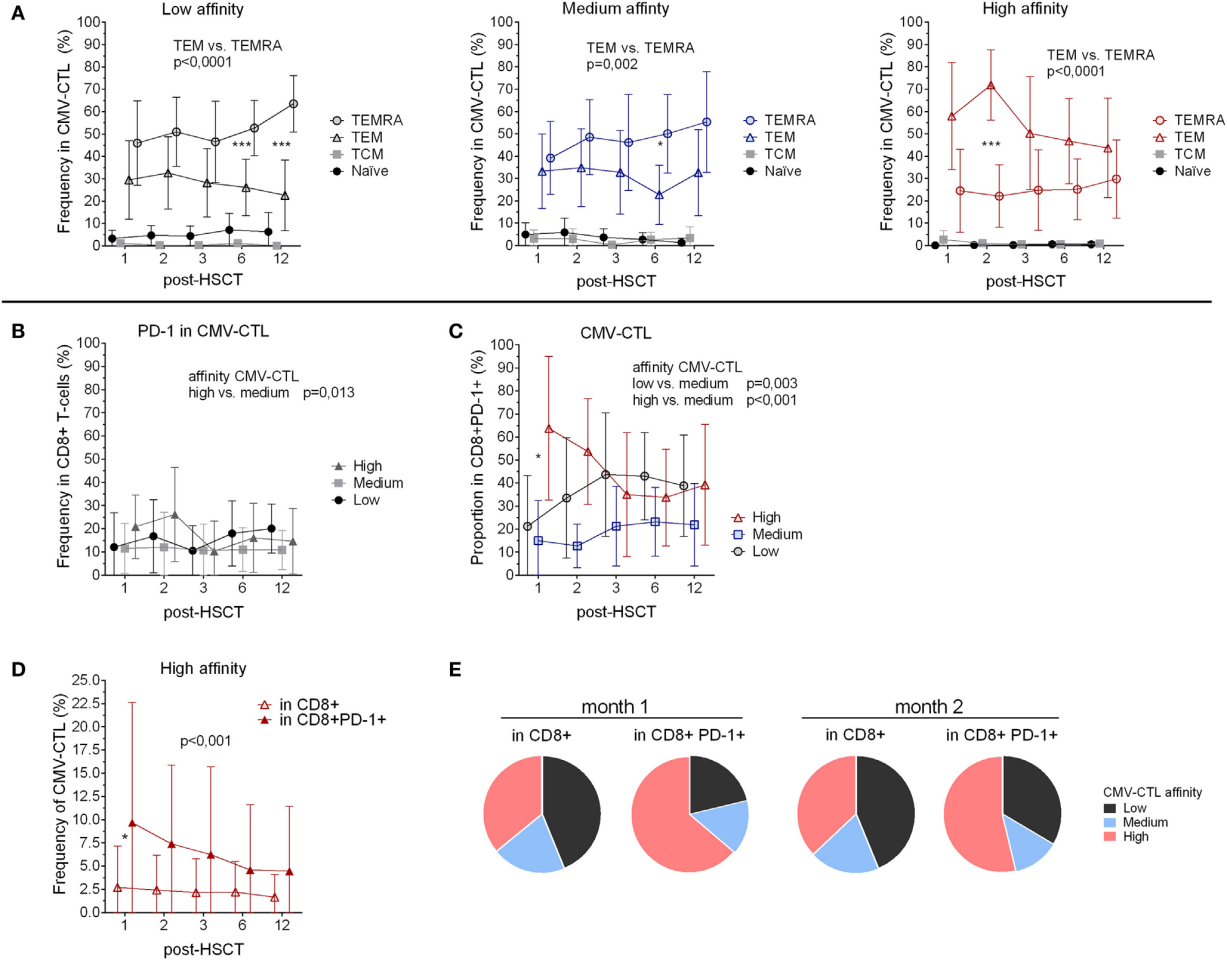
Memory phenotype and PD-1 expression of the cytomegalovirus(CMV)-specific CD8+ cytotoxic T-lymphocytes (CMV-CTL) with different T-cell receptor affinities over time post-hematopoietic stem cell transplantation (HSCT). **(A)** Memory phenotype of the different CMV-CTL subpopulations. **(B)** PD-1 expression in the different CMV-CTL subpopulations **(C)** Proportion of the high/medium/low-affinity CMV-CTL in the CD8+PD-1+ T-cell population. **(D)** Comparison of the high-affinity CMV-CTL frequency between the total CD8+ T-cell population and the CD8+PD-1+ T-cell population. Mean and SD are represented. Two-way ANOVA was used to detect differences between two groups over time. *p < 0.05, **p < 0.01, ***p < 0.001. **(E)** Pie charts with the proportion of high/medium/low-affinity CMV-CTL the total CD8+ T-cell population and the CD8+PD-1+ T-cell population at month 1 and 2 post-HSCT.

Analyzing the CD45RA and CCR7 expression of the three different CMV-CTL affinity populations, we determined the presence of different memory phenotypes within the CMV-CTL overtime post-HSCT. Overall, the three different classes of CMV-CTL presented a low proportion of naïve (CD45RA+ CCR7+) and TCM T-cells. Low- and medium-affinity CMV-CTL demonstrated a predominant TEMRA phenotype while high-affinity CMV-CTL showed a predominant TEM phenotype (*p* < 0.001) overtime post-HSCT (Figure 2A). The TEMRA population in the low- and medium-affinity CMV-CTL presented an increase mean from 45.9 to 63.6% and from 39.1 to 55.3%, respectively. This increase was significant between months 1 and 6 in the medium-affinity CMV-CTL (*p* = 0.019). Reversely, the TEM population of the high-affinity CMV-CTL decreased over time (mean from 57.9 to 43.7%) and significant between 2 and 6 months post-HSCT (*p* = 0.037) (Figure S3A in Supplementary Material).

We analyzed the CMV-CTL affinity classes within the different memory subsets and observed that high-affinity CMV-CTL were found in a significantly higher proportion as compared to the medium and low CMV-CTL classes in the TEM CD8+ T-cell subset (*p* < 0.001 and 0.005). Conversely, the TEMRA population was preferentially enriched in CMV-CTL with low-affinity TCR (*p* = 0.001) (Figure S3B in Supplementary Material).

To summarize, phenotypic differences were observed within the CMV-CTL population depending on the TCR affinity, with high-affinity CMV-CTL displaying predominantly a TEM phenotype while low and medium-affinity CMV-CTL exhibited a TEMRA profile.

### PD-1 Expression Correlates With Memory Phenotype and TCR Affinity of CMV-Specific CD8+ T-Cells Post-HSCT

To further characterize the different classes of CMV-CTL during the reconstitution, we examined PD-1 expression. CD8+PD-1+ T-cells displayed a memory phenotype similar to the total CD8+ population with a predominant TEM phenotype, and an increasing TEMRA phenotype (mean from 28.5 to 37.4%, *p* < 0.039) between months 2 and 6 (Figure S3C in Supplementary Material).

Despite being a marker of exhaustion, PD-1 expression also identifies antigen-experienced T-cells (45). We, therefore, investigated the correlation between PD-1 expression and the different affinities of the CMV-CTL. PD-1 expression was significantly higher among cells with high-affinity TCR compared to the medium-affinity CMV-CTL (*p* = 0.013) post-HSCT, while no difference in PD-1 expression between low- and high-affinity CMV-CTL was observed (Figure 2B). Within the CD8+PD-1+ T-cell population, the proportion of the different CMV-CTL classes was found to be different as compared to the proportion described above in the total CD8+ T-cell population (Figure 1G): PD1+ high- and low-affinity CMV-CTL were found in higher proportion as compared to medium-affinity CMV-CTL (*p* < 0.001 and 0.003, respectively, Figure 2C; Figure S3D in Supplementary Material). Additionally, at month 1 post-HSCT only, PD1+ CMV-CTL with high-affinity TCR were found at significant higher proportion as compared to PD1+ low-affinity CMV-CTL (*p* = 0.012). Altogether, the frequency of CMV-CTL seemed to be higher in CD8+ T-cells expressing the PD-1 marker: high-affinity CMV-CTL appeared to be at higher frequency in CD8+PD-1+ T-cells as compared to the total CD8+ T-cell population (*p* < 0.001), and at 1 month post-HSCT (*p* = 0.036, Figure 2D). To a lesser extent, low-affinity CMV-CTL was as well found at higher frequency in the CD8+PD1+ subset as compared to the total CD8+ population (*p* = 0.049, Figure S3E in Supplementary Material). Consequently, the CMV-CTL with high-affinity TCR were found at higher proportion in the CD8+PD-1+ population as compared to the medium and low-affinity CMV-CTL and in the total CD8+ population in the early months post-HSCT (Figure 2E).

To summarize, CMV-CTL were found at higher frequency in the CD8+PD-1+ T-cells as compared to the total CD8+ T-cells. In the CD8+PD-1+ T-cell population, CMV-CTL with high-affinity TCR were found mostly early post-HSCT at higher proportion as compared to CMV-CTL with low- and medium-affinity TCR.

### Analysis of Factors Affecting CMV-Specific CD8+ T-Cells

**Figure 3:**
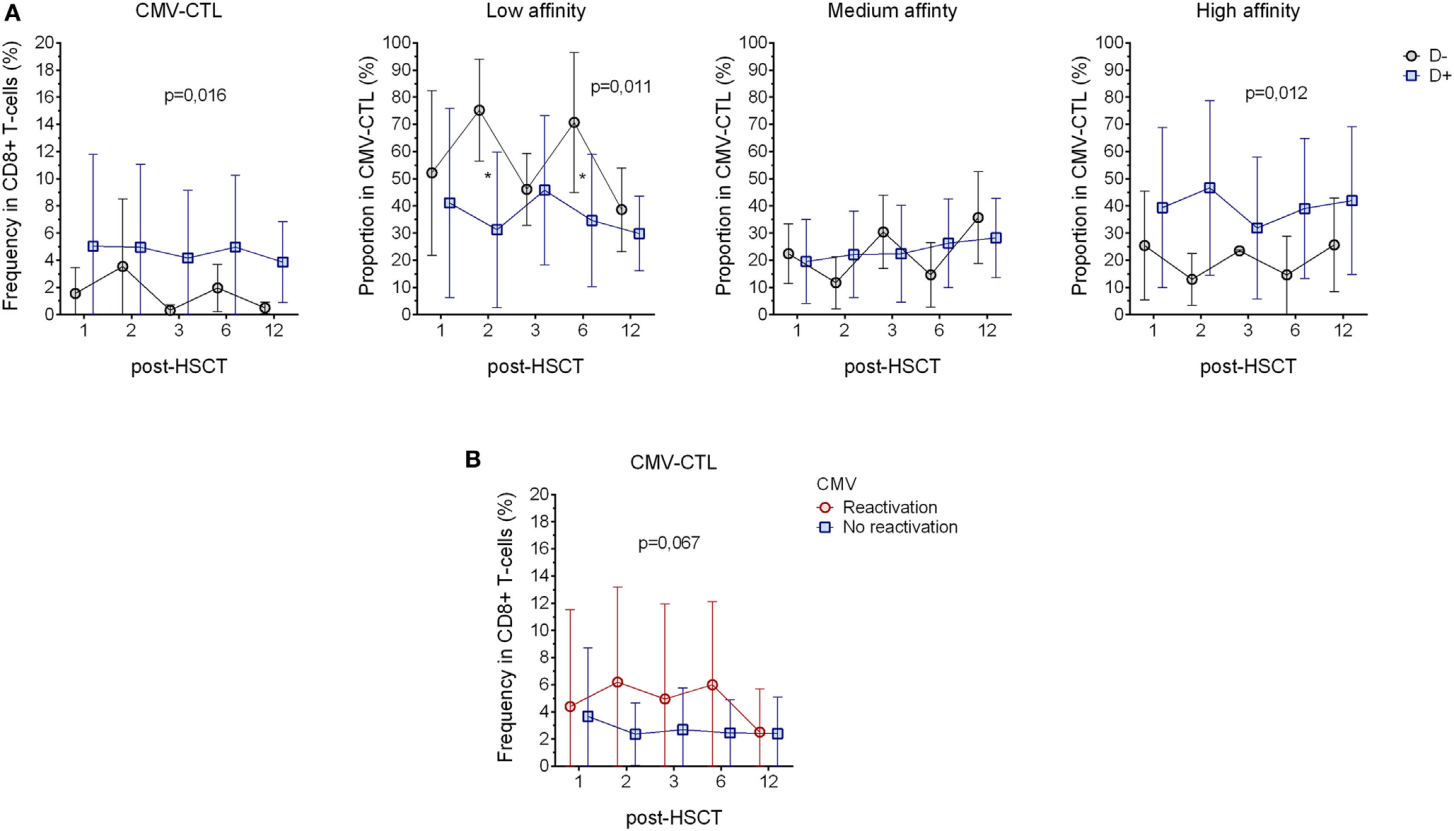
Correlation with the donor serostatus and cytomegalovirus (CMV) reactivation. **(A)** Comparison of CMV-CTL frequency, low-, medium-, and high-affinity proportion between patients with donor presenting different CMV-serological status (D+: blue square and D−: black circle) **(B)** Analysis of CMV-CTL in between patients diagnosed with CMV reactivation (red circle) and patients who did not present sign of reactivation (blue square). Mean and SD are represented. Two-way ANOVA was used to detect differences between two groups over time. *p < 0.05.

To understand the clinical impact of the CMV-CTL classes based on their TCR affinity, we evaluated the correlation between clinical data and the findings mentioned above, i.e., CMV serostatus, GVHD and CMV reactivation, CMV-CTL affinity classes, and PD-1 expression. The CMV-serological status of the donor and the CMV reactivation appeared to correlate to the total frequency of CMV-CTL, since patients with a donor presenting a CMV-positive serostatus (D+, *n* = 16) presented a higher frequency of CMV-CTL as compared to the patients with a CMV-negative donor (D−, *n* = 7). Interestingly, when looking at the proportion of the different classes of CMV-CTL, we observed that the D+ patients presented a significant higher proportion of CMV-CTL with high-affinity (*p* = 0.012), while in the D− patients, CMV-CTL expressed predominantly low-affinity TCRs (*p* = 0.011) (Figure 3A). No difference was observed in the proportion of medium-affinity CMV-CTL between the D− and D+ patient group. Out of the five patients with a negative serological status, only four patients presented measurable CMV-CTL frequencies, therefore, no correlation analysis between the recipient serological status and the proportion of the different CMV-CTL could be performed.

11 out of 23 patients were diagnosed for CMV reactivation during the 12 months post-transplantation. Patients diagnosed for CMV reactivation were found to have a higher frequency of CMV-CTL, yet, due to the small number of patients where there were no significant differences between the groups (*p* = 0.067, Figure 3B). As reported previously (2), we showed that CMV reactivation shapes the memory phenotype of the CD8+ T-cells, as in patients with CMV reactivation, CMV-CTL presented a significant higher TEM phenotype (*p* = 0.001) over time post-HSCT as compared to patients without CMV reactivation. On the other hand, patients with CMV reactivation showed a lower frequency of TEMRA CD8+ T-cells (*p* = 0.005, Figure S4A in Supplementary Material). PD-1 expression has also been described to be rapidly expressed after antigen exposure and is associated with an impairment of the immune system associated with chronic viral infections (46, 47), but no such observation was made in our study as we could not see differences in PD-1 expression between the patients with and without CMV reactivation (Figure S4B in Supplementary Material).

**Figure 4:**
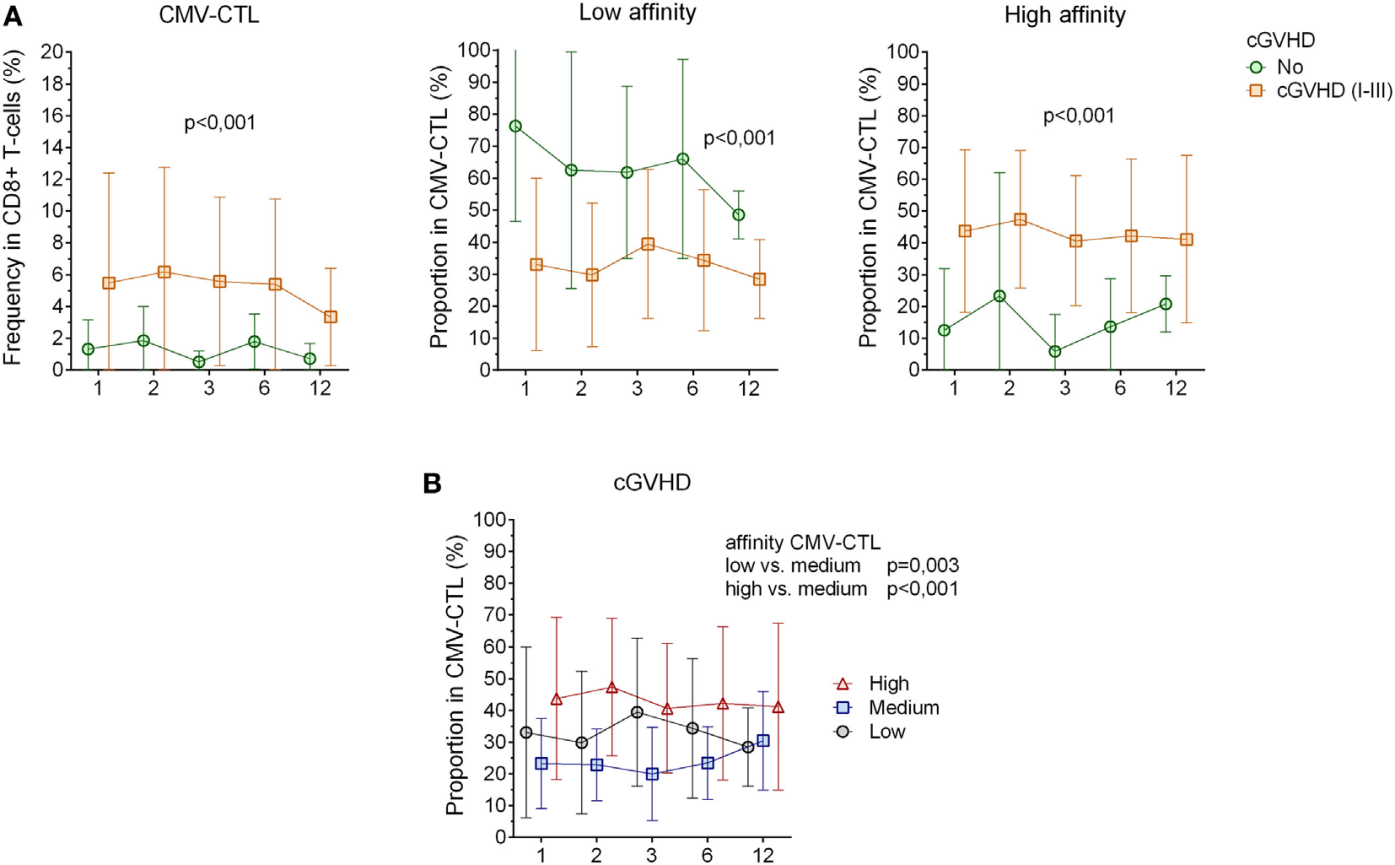
Correlation between cytomegalovirus(CMV)-specific CD8+ cytotoxic T-lymphocytes(CTL) affinity and cgraft-versus-host disease(GVHD). **(A)** CMV-CTL frequency, low and high-affinity proportion correlated with cGVHD. Green circle: no cGVHD, orange square: cGVHD (I–III) **(B)** Proportion of high/medium/low-affinity CMV-CTL in patients presenting clinical symptoms of cGVHD (I–III). Black circles: low-affinity CMV-CTL, blue squares: medium-affinity CMV-CTL, red triangles: high-affinity CMV-CTL. Mean and SD are represented. Two-way ANOVA was used to detect differences between two groups over time.

No significant correlation between clinically relevant grade (II–IV) of acute GVHD (8/23) and our immunological characterization could be determined. However, patients with chronic GVHD (13/23, Table 1) had a higher frequency of CMV-CTL over time post-HSCT as compared to patients who did not present any sign of cGVHD (*p* < 0.001). High-affinity CMV-CTL were found in higher proportion in patients with cGVHD and conversely, low-affinity CMV-CTL proportion was higher in patients who did not present sign of cGVHD (*p* < 0.001, Figure 4A). Within the cGVHD patients’ group, CMV-CTL with high-affinity TCR was the dominant proportion of CMV-CTL, yet a significant difference was only observed with the medium-affinity CMV-CTL proportion (*p* < 0.001, Figure 4B). In patients diagnosed with CMV reactivation, 8/11 (72.7%) were diagnosed with cGVHD, while among the 12 patients that did not present any CMV reactivation, five of them (41.6%) presented cGVHD symptoms, but no correlation could be made between CMV reactivation and chronic GVHD in this small study cohort. Also, no correlation between the death of patients and the different CMV-CTL based on TCR affinity could be found possibly due to the limited number of patients included for this study.

To summarize, in patients with CMV reactivation, CMV-CTL showed a TEM phenotype over time post-HSCT. CMV-CTL with high-affinity TCR was significantly found at higher proportion in CMV-D+ patients and in patients diagnosed with cGVHD.

## Discussion

Cytomegalovirus-specific immune responses are crucial post-HSCT; transplant recipients with poor reconstitutions of CMV-specific T-cell response are more prone to develop CMV disease (3). While antiviral treatments remain the standard therapy for CMV infection post-HSCT, the clinical use of adoptive CMV-specific T-cell therapy has become a successful alternative over the past 25 years (19, 22, 23, 48). In this paper, we show the existence of different proportion of CMV-CTL based on their TCR affinity. This description was only possible by using three different HLA-A*02:01 CMV_NLVPMVATV_ tetramers. To our knowledge, we reported here for the first time the ability of the mutant q226a tetramer to enable the detection of high-affinity CMV-CTL independent of the CD8-coreceptor interaction.

The current viewpoint on CMV-mediated immune responses is that high- and low-affinity T-cells have specific as well as overlapping functions, which partly shapes the diversity of the immune response during infection and disease (49, 50). In healthy individuals, high- and low-affinity antigen-specific CTL would preferably function together to maintain a broad TCR diversity, while eliciting an efficient and targeted immune response (44). High-affinity CTL is more likely to arise from clonal expansion of CD8+ T-cells and is, therefore, more suitable for ACT (51). In the context of HSCT, a245v mutant CMV tetramer T-cells was used in D+R+ patients to study the different avidities of CTL presenting similar phenotype and functional activity, but with different TCR repertoire from donor origin exclusively (52), however, the characterization of TCR affinity was not part of this study.

Affinity of CMV-specific T-cells has been indirectly described using blockade of the MHC class I interaction with an antibody directed against CD8 (51); different CMV-CTL clones reacted variably to infected cells. Their cytotoxic activity appeared to be influenced by the abrogation of CD8 binding (49). In our study, we observed that the same population of virus-specific T-cells could have different memory phenotypes, since the high-affinity CD8+ T-cells were mostly effector memory T-cells as compared to the medium- and low-affinity CD8+ T-cells. Correlation between high-affinity TCR and effector phenotype has been described earlier and high-affinity T-cells may detect early viral antigens during low-level infection (53–56). Similar observations were made by Price DA et al. in which they showed that distinct CMV-CTL clones exhibited different memory phenotypes by using a D227K/T228A mutant tetramer to target high affinity CD8+ T-cells (57). Furthermore, high-affinity clones have been found to dominate the adaptive immune response at the peak of the effector response (58) and enriched in large frequency in the memory pool, with T-cells expressing high-affinity TCR during secondary response (59). Together, this observation suggests that long-lived protective T-cell responses may be fueled by high-affinity CD8+ T-cells with an effector memory phenotype (60, 61).

Programmed death-1 expression has been previously associated with post-HSCT relapse in patients with acute myeloid leukemia, but does not exert a deleterious effect on the functionality of T-cells, based on their ability to produce cytokines in response to polyclonal stimulation (62). In addition, the increase expression of PD-1 in T-cells has also been linked to persistent CMV infection and GVHD (46, 47). However, no significant differences in the PD-1 expression pattern were observed in our study over time post-HSCT, certainly due to the few number of patients diagnosed with CMV disease (*n* = 3). Conversely, we demonstrate here a correlation between PD-1 expression and CMV-specific CD8+ T-cells response, i.e., the CMV-CTL population with high-affinity TCR was found at higher frequency among the PD-1 positive T-cells compared to the frequency observed in total CD8+ T-cells. Considering that the PD-1+CD8+ T-cells from HSCT recipients mainly express an effector memory phenotype, the expression of PD-1 is not necessarily associated with T-cell dysfunction. CMV-specific response has its particularity; unlike antigen-specific T-cells in other chronic viral infections, i.e., LCMV, HIV, HCV, or HBV, where PD-1 expression is rather high (63–66), CMV-specific T-cells seem to express PD-1 at a much lower levels (approximately 5–12%). CD8+PD-1+ T-cells specific for CMV epitopes may preserve the full spectrum of their functional capacity by producing cytokines albeit without cytotoxic or proliferative potential (67). CD8+PD-1+ T-cells are very likely to have undergone antigen-driven clonal expansion in the HSCT recipients during immune reconstitution, and are possibly associated with recognition of private epitopes, such as in advanced cancers (68, 69). PD-1 expression may in fact prevent the exhausted/antigen-experienced T-cells from being programmed for terminal differentiation and excessive proliferation (70). It was shown that virus-specific CD8+ tumor-infiltrating lymphocytes expressing PD-1 were not impaired, but described as newly antigen experienced (71). In the tumor environment, high-affinity CD8+ T-cell clone presented a significant anti-tumor response with a lower expression of PD-1, while the low-affinity clone increasingly expressed several co-inhibitory molecules (72).

Donor CMV serostatus and conditioning of patients can impact the outcome of HSCT as well as the reconstitution of the CMV-specific immune response post-HSCT (73–77). CMV-CTL from the donors have been described to be multifunctional as defined by production of IFN-γ and TNF-α, co-expression of the degranulation marker CD107a and macrophage inflammatory protein-1β (78). Similar observations were made in this study as patients with D+ CMV serostatus presented a higher frequency of CMV-CTL most likely from donor origin, since the chimerism analysis of the CD3+ cells indicated that the CMV-CTL were of donor origin, at least at the later time points post-HSCT.

Association between viral reactivation, its specific immune response, and GVHD have been studied extensively and several studies showed that GVHD treatment increases the risk of CMV replication, whereas the data showing the reverse relationship remain controversial (79–81). One strong argument against this reverse relationship would be the use of CMV-CTL ACT for restoring anti-viral T-cell immunity that has been clinically applied over the past 25 years without showing any increased risk to induce cGVHD (23, 82, 83). Isolation with MHC tetramer of CMV-CTL for ACT purposes have, to our knowledge, previously been used on non-mutated tetramers, therefore, the affinity of the ACT product has never been specifically characterized before infusion (18, 84–87). In this study, we give a more general insight on possible improvement of the ACT protocols as the selection of CD8+ T-cells with specific TCR affinity may be more effective in clearing infected or tumor cells and conferring a better long-term protective memory. The affinity of the interaction between the TCR and the peptide bound to the MHC molecule is known to play a major role in the efficacy of ACT and it is suggested that high-affinity T-cells with TCR independent of CD8 binding to the MHC may be beneficial for clinical applications, including ACT (88). Thus, assessment and selection of antigen-specific T-cells with optimal affinity/reactivity from a heterogeneous T-cell population may help in the development to improve the T-cell product and thereby enhance clinical efficacy. This selection based on TCR affinity has to be careful as we observed that patients with cGVHD exhibit a higher frequency of CMV-CTL and a significantly higher proportion of CMV-CTL with high-affinity TCR, while low-affinity CMV-CTL were at higher proportion in patients who did not present any symptoms of cGVHD. Interestingly, previous studies have suggested that T-cells with the highest affinity for its antigen were the most alloreactive (89, 90). A recent clinical trial using engineered T-cells expressing an affinity-enhanced TCR for tumor-associated antigens demonstrated unpredictable off-target due to recognition of an unrelated peptide resulting in the death of two treated patients (91).

This study is limited by its small cohort size and the low number of T-cell events due to the HSCT conditioning. This study was also mostly composed by sibling donors and RIC transplants cohort as we selected patients not treated with ATG in order to increase the chance to observe CMV-CTL at sufficient number to further phenotype and characterize them. Therefore, further investigation using the CMV tetramers identifying TCRs with different affinities in a larger and more heterogeneous cohort and in healthy individuals would allow to explore the function of the different CMV-CTL subpopulations. This would give a more descriptive picture and clear insight of the TCR affinity implication following HSCT procedure. Cell expansion of the different CMV-CTL subpopulations would give a more comprehensive characterization of their proliferation potentials that could also be tailored for adaptive cellular immunotherapies targeting viral epitopes. It would also be interesting to see whether blockade of the PD-1 pathway can differently affect the expansion and function of the different subpopulations of CD8+ T-cells in patients undergoing HSCT.

In conclusion, we have investigated the reconstitution of CMV-CTL with different TCR affinities in HSCT patients by using three different tetramers. The q226a mutant tetramer identifies exclusively high-affinity CMV-CTL that does not require the CD8 co-receptor for MHC-TCR interaction. High-affinity CMV-CTL could be found at higher proportion in experienced CD8+PD-1+ population and presented an effector phenotype in contrast to the CMV-CTL with low- and medium-affinity TCR that exhibited a dominant TEMRA phenotype in patients after HSCT. Patients with chronic GHVD exhibited a higher proportion of high-affinity CMV-CTL, while patients who did not present any sign of cGVHD showed a dominant proportion of low-affinity CMV-CTL. Further studies on the TCR affinity of CMV-CTL after HSCT are needed to elucidate their functions and possible clinical implication post-HSCT and for ACT.

## Ethics Statement

Ethical approval was obtained from the regional review board of ethics in research of Karolinska Institutet (Stockholm Ethical Committee South 2010/760-31/1). Written informed consent was received from participants prior to inclusion in the study.

## Author Contributions

TP conceived, designed, performed, and analyzed the experiments, interpreted the results, performed statistical analysis, and wrote the paper. RA-R conceived and designed the tetramers, interpreted and analyzed the experiments and results, and wrote the paper. MRemberger interpreted the results and performed statistical analysis. MRao and X-HL interpreted the results and wrote the paper. AN and AL performed the experiments. IE, OR and MM conceived, designed the experiments, and wrote the paper. All authors participated in final approval of the manuscript.

## Conflict of Interest Statement

The authors declare that the research was conducted in the absence of any commercial or financial relationships that could be construed as a potential conflict of interest. The reviewer EA and handling Editor declared their shared affiliation.

## Supplementary Material

Figure S1 Phenotype of the CD8+ T-cell population post-HSCT. **(A)** Representative flow cytometry plots from one patient showing the gating strategies to identify the different cell subsets. **(B)** Gating strategy to separate the different subset of CMV-CTL based on the tetramer staining. **(C)** Donor chimerism on CD3+ cells in peripheral blood post-HSCT expressed as percentage of donor DNA medians are represented. **(D)** CD8+ T-cells reconstitution post-HSCT. **(E)** Memory phenotype composition of the CD8+ T-cells based on CD45RA and CCR7 markers. **(F)** Memory phenotype of the CD8+ T-cells in individual patients followed post-HSCT. **(G)** PD1 frequency in CD8+ T-cell population post-HSCT. **(H)** PD1 mean fluorescence intensity (MFI) in the CD8+ T-cell subsets. Means are represented for **(E,G,H)**. Wilcoxon matched-pairs signed rank test or Friedman test to detect differences across between two matched timepoints or repeated matched time points after HSCT. **p* < 0.05, ***p* < 0.01, ****p* < 0.001.

Figure S2 Representative flow cytometry plots from three different patients illustrating the different configurations of CMV-CTL proportion based on the TCR affinity. Configuration 1 represents a minor proportion of high affinity CMV-CTL, configuration 2 represents an equal proportion between CMV-CTL with different TCR affinity, and configuration 3 represent a dominant proportion of high affinity CMV-CTL.

Figure S3 Memory subsets and PD1 expression of the different CD8+ T-cell affinity subgroups CMV tetramer reactive CD8+ T-cells. **(A)** Low- and medium-affinity CMV-CTL presented a more TEMRA profile over the time post-HSCT. High affinity CMV-CTL presented a dominant but decreasing TEM phenotype over time post-HSCT. **p* < 0.05. **(B)** Proportion of CMV-CTL with different TCR affinities in the TEM and TEMRA memory subsets post-HSCT. Means and SD are represented. **(C)** Memory phenotype composition of the CD8+ PD-1+ T-cells based on CD45RA and CCR7 markers. Means are represented. **(D)** Low-, medium-, and high-affinity CMV-CTL frequency in the CD8+ PD-1+ T-cells. **(E)** Comparison of low-affinity CMV-CTL frequency in CD8+ T-cells and CD8+ PD-1+ T-cells. Means and SD are represented. Wilcoxon matched-pairs signed rank test or Friedman test to detect differences across between two matched timepoints or repeated matched time points after HSC in **(A)** and two-way ANOVA was used to detect differences between two groups over time in **(B,E)**.

Figure S4 Clinical correlation **(A)** CD8+ T-cell memory phenotype correlates to CMV reactivation. Patients with CMV reactivation presented a higher frequency of TEM T-cells and a lower frequency of TEMRA T-cells. **(B)** Expression of PD-1 did not correlate with CMV reactivation over time post-HSCT. Means and SD are represented and two-way ANOVA was used to detect differences between two groups over time.
