# Supplementary material for: Cytomegalovirus-Specific CD8+ T-Cells With Different T-Cell Receptor Affinities Segregate T-Cell Phenotypes and Correlate With Chronic Graft-Versus-Host Disease in Patients Post-Hematopoietic Stem Cell Transplantation
Source: Front Immunol. 2018 Apr 10;9:760. doi: 10.3389/fimmu.2018.00760 (PMC5903031; doi:10.3389/fimmu.2018.00760)
Supplement: Supplementary file 5 [file table_1.PDF]

Supplementary Table 1: Patients information

ID	Gender	Disease	Donor	Age	CMV serology		CMV infection/ reactivation	CMV treatment (days post-HSCT)	GVHD grade (days of onset post-HSCT)		DLI (days post-HSCT)	Cause of Death (days post-HSCT)
					Rec	Don			acute	chronic		
**SCT 1**	F	MDS	brother	45	-	+	N	N	1 (34)	1 (117)	N	
**SCT 2**	F	MDS	sister	64	+	-	Y	GCV (111)	3 (107)	1 (198)	N	Sec Mal (1228)
**SCT 3**	F	EBV driven polyclonal lymphoprolif disease	sister	22	+	+	N	N	0	0	N	
**SCT 4**	M	ALL	sister	27	+	-	Y	N	2 (18)	0	N	
**SCT 5**	M	MDS	brother	60	+	+	N	N	2 (17)	0	N	
**SCT 6**	F	Myelofibrosis	brother	63	+	+	Y	GCV (124)	3 (19)	0	N	aGVHD (166)
**SCT 7**	F	Myelom	brother	40	+	+	Y	N	0	3 (87)	N	
**SCT 8**	F	Severe aplastic aneamia	unrelated	53	-	-	N	N	4 (21)	0	Y (106)	aGVHD / IFI (140)
**SCT 9**	F	Myelom	sister	50	-	+	N	N	0	0	N	
**SCT 10**	F	ALL ph+	sister	61	+	+	Y	N	1 (25)	3 (616)	N	
**SCT 11**	F	T-PLL	unrelated	61	+	+	Y	N	1 (45)	1 (965)	N	
**SCT 12**	M	CML	brother	38	-	-	N	N	1 (26)	0	N	
**SCT 13**	F	T-lymphoblastic lymphoma	unrelated	35	+	+	N	N	1 (30)	0	N	Relapse / viral infection (188)
**SCT 14**	M	AML	brother	29	+	+	Y	N	1 (65)	1 (137)	N	
**SCT 15**	M	AML	brother	54	+	-	Y	N	2 (17)	3 (211)	N	
**SCT 16**	F	AML	sister	36	+	-	N	N	2 (29)	0	N	
**SCT 17**	M	AML	brother	57	+	+	Y	VCV (111)	3 (81)	3 (363)	N	
**SCT 18**	F	AML	brother	72	+	+	N	N	0	1 (95)	N	
**SCT 19**	F	MDS-AML	brother	61	+	-	Y	N	1 (47)	0	N	Relapse / bacterial infection (237)
**SCT 20**	F	NHL	sister	62	+	+	N	N	1 (20)	1 (222)	N	
**SCT 21**	F	MDS	sister	51	+	+	Y	N	1 (49)	1 (98)	N	Relapse (536)
**SCT 22**	M	MDS	sister	64	-	+	N	N	1 (44)	3 (145)	N	
**SCT 23**	F	ALL	sister	40	+	+	N	N	0	1 (98)	N	

DLI: donor lymphocyte infusion, MDS: myelodysplastic syndromes, EBV: Epstein-Barr virus, ALL (ph+): acute lymphoblastic leukemia (Philadelphia chromosome positive), T-PLL: T-cell-prolymphocytic leukemia, CML: chronic myeloid leukemia, AML: acute myeloid leukemia, NHL: Non-Hodgkin lymphoma, N/Y: No/Yes, GCV: ganciclovir, VCV: Valganciclovir, aGVHD: acute graft-versus-host disease, Sec Mal: secondary malignancy, IFI: invasive fungal infection
